# LncRNA GSTM3TV2 Promotes Cell Proliferation and Invasion via miR-597/FOSL2 Axis in Hepatocellular Carcinoma

**DOI:** 10.1155/2021/3445970

**Published:** 2021-08-17

**Authors:** Yuting Hu, Wei Qiu, Zhijun Kong, Siyuan Wu, Yi Liu, Chunfu Zhu, Xihu Qin

**Affiliations:** ^1^Department of Hepatobiliary Surgery, The Affiliated Changzhou NO.2 People's Hospital of Nanjing Medical University, Changzhou, Jiangsu Province 213003, China; ^2^Department of Hepatobiliary and Pancreatic Surgery, The First Hospital of Hanbin District, Ankang, Shanxi Province 725000, China

## Abstract

Mounting evidence has recently shown that role of long noncoding RNA is critical in many human cancers. lncRNA GSTM3TV2 was first proven to play a vital role in pancreatic cancer. However, the mechanism of lncRNA GSTM3TV2 in hepatocellular carcinoma (HCC) is still uncovered. Here, we object to distinguish the expression of lncRNA GSTM3TV2 and reveal its mechanistic relationship with HCC. We observed that the expression of lncRNA GSTM3TV2 and FOSL2 were upregulated in HCC. Knockdown of lncRNA GSTM3TV2 significantly inhibited cell proliferation. Meanwhile, the migration and invasion of HCC cells were greatly decreased by the downregulated lncRNA GSTM3TV2. The luciferase reporter assays showed that lncRNA GSTM3TV2 could be directly bound to miR-597, and the level of miR-597 was also decreased in the tumor tissues. lncRNA GSTM3TV2 could stabilize FOSL2 expression, resulting in the oncogenic properties of lncRNA GSTM3TV2 in HCC. Our study indicated the oncogenic activities of lncRNA GSTM3TV2 and emphasized the role of the miR-597/FOSL2 signaling pathway.

## 1. Introduction

Hepatocellular carcinoma (HCC) is now becoming a main global problem, because it is one of the most mortal cancer all over the world [1]. The high mortality is due to the late stage of presentation in the majority of cases and the limited treatment options in that situation where beneficial surgery is no longer possible [2]. This late presentation is due in a large part to the absence of symptoms in the early stages of the disease and the lack of diagnostic biomarkers and other methods for detecting HCC in the early stage [3]. Potentially effective interventions, like surgery and liver transplantation, are only suitable for the early stage. The options of treatment should be loco-regional or systemic. For systemic treatment, chemotherapy and targeted therapy options are clinically approved for treating locally advanced or metastatic HCC, which extend survival by only some months in patients [4]. Given the dismal landscape of therapeutic options if diagnosis is made late, therefore, there is an urgent essential for a fine-grained molecular landscape of HCC from which to discover clinically relevant early diagnostic and prognostic biomarkers and to develop beneficial precision therapies [5].

Long noncoding RNAs (lncRNAs) are a kind of functional transcripts, whose length are more than 200 nucleotides participating in biological functions [6], including regulation of basal transcription machinery [7], gene-specific transcription [8], translation [9], and epigenetic modifications [10]. Today, increasing lcnRNAs have been reported to take part in promoting or suppressing the pathogenesis of cancer [6], including regulating cell cycle [11], survival [12], apoptosis [13], invasion, and metastasis [14] in cancers. Remarkably, some lncRNAs are found to be important roles in the efficacy of anticancer therapies in cancer, including HCC. For example, HULC could prevent miR-107 from binding E2F1 transcription factor, enhancing expression of SPHK1 and angiogenesis [15]. SNHG6-003, another upregulated lncRNA in HCC, promotes the proliferation of HCC cell lines [16]. Meanwhile, lncRNA-ATB can inhibit the miR-200 family, which plays an important role in tumor invasion [17].

A recent study showed that GSTM3TV2 (Homo sapiens glutathione S-transferase mu 3, transcript variant 2) could promote resistance of gemcitabine in pancreatic cancer by sponging let-7 competitively to upregulate the LAT2 and OLR1 expression [18]. And the role of lncRNA GSTM3TV2 in HCC is still unknown. So, we measured the level of lncRNA GSTM3TV2 expression in HCC, and we found that lncRNA GSTM3TV2 was upregulated in HCC tissues and cell lines. We assumed that lncRNA GSTM3TV2 may play an important role in HCC and act as the ceRNA role to regulate proliferation and migration in HCC cell lines. Then, we investigated that lncRNA GSTM3TV2 could promote HCC cell proliferation and migration by upregulating FOSL2 expression through competitively sponging miR-597. Thus, the high level of GSTM3TV2 would be a worse prediction. In a word, our data revealed that GSTM3TV2 could act as a new prognostic marker and a therapeutic target in HCC.

## 2. Materials and Methods

### 2.1. Tumor Tissues Collection

30 pairs of hepatocellular carcinoma tissues samples, as well as their adjacent normal tissues, were obtained from the Affiliated Changzhou NO.2 People's Hospital. The information was summarized in Table 1. All the pathologically and histologically HCC patients have been stored at -80°C before the study. All methods were achieved according to the guideline approved by the Ethics Committee of Affiliated Changzhou NO.2 People's Hospital. All patients were given informed consent and written informed consent was obtained.

### 2.2. Cell Culture

Human HCC cell lines (LO2, SMMC-7721, MHCC97-H, HepG2, and Hep3B) were cultured in DMEM medium (Invitrogen, USA), containing with 10% fetal bovine serum (Gibco, USA). All the above cells were cultured at 37°C.

### 2.3. Real-Time Quantitative PCR

The total RNA and miRNA Isolation was carried out by using QIAGEN Rneasy Mini kit (Invitrogen, USA) and QIAGEN miRNeasy Mini Kit (Invitrogen, USA) according to the manufacturer's instructions. Quantitative PCR analysis of miR-597 was performed by using commercial TaqMan microRNA assays (Invitrogen, USA).

### 2.4. MTT Assay

4 × 10^3^ cells were plated into 96-well plates to detect cell viabilities and cultured 18–24 h before transfecting with vectors, mimics, or plasmids. Then, 24 h, 48 h, and 72 h later, an MTT assay was performed to check the cell viabilities at 570 nm by using a Quant Universal Microplate Spectrophotometer (BioTek, USA).

### 2.5. Vector Construction

Complementary DNA encoding GSTM3TV2 was synthesized and subcloned into the pcDNA3.1(+) vector (Invitrogen) according to the manufacturer's instructions. The pcDNA3.1-GSTM3TV2 construct containing point mutations was synthesized by GENEWIZ (Jiangsu, China) and named pcDNA3.1-GSTM3TV2-Mut. The miR-597 binding region in either lncRNA-GSTM3TV2 or lncRNA-GSTM3TV2-Mut was amplified using PCR and subcloned into the pmirGLO vector (Promega, Madison, WI, USA) for use in a luciferase reporter assay.

### 2.6. Transwell Assay

Cell suspension was added into transwell chamber inserts (Millipore, USA) and added with matrigel. 24 h later, cells were stained and pictures were taken to measure invasion assays in accordance with the manufacturer's instructions as described previously.

### 2.7. Wound-Healing Migration Assay

HCC cells were cultured in six-well plates to perform migration assays. A 200 *μ*l pipette tip was used to scratch to generate a linear gap. After 24 h, we used the microscope to take pictures and measured the width (*W*) of the scratch wound. The rate of close distance of the wounds was calculated. All measurements were carried out three times.

### 2.8. Luciferase Assay

After construction, the pGL/Luc-lncRNA GSTM3TV2-wild type or mutation plasmids were transfected into the cells (SMMC-7721 and HepG2), as well as with miR-597 mimics, and ASO (antisense oligonucleotide)-miR-597 or NC (negative control). Cell lysates were detected by a Luciferase Reporter System (Promega, USA) after 48 h.

### 2.9. Western Blot

The tissue and cellular protein were extracted by using RIPA lysis buffer. After centrifuge, the concentration of protein was checked by BCA kit. Western blot was performed in accordance with the standard protocol anywhere. FOSL2, Vimentin, and GAPDH antibodies were obtained from Cell Signal Technology (CST, USA). The bands were obtained from Image Lab after adding chemiluminescent substrate (ECL; Millipore) to visualize. The Image Lab software was used to analyze results.

### 2.10. Statistical Analysis

Statistical analyses were performed by SPSS IBM 20.0. The *P* values were determined by using *t*-test (student's *t*-test) or ANOVA (Analysis of Variance). All data in the graphs are showed as mean ± SD. (^∗^*P* < 0.05; ^∗∗^*P* < 0.01; ^∗∗∗^*P* < 0.001; ^∗∗∗∗^*P* < 0.0001).

## 3. Results

### 3.1. lncRNA GSTM3TV2 in HCC Was Upregulated

**Figure 1:**
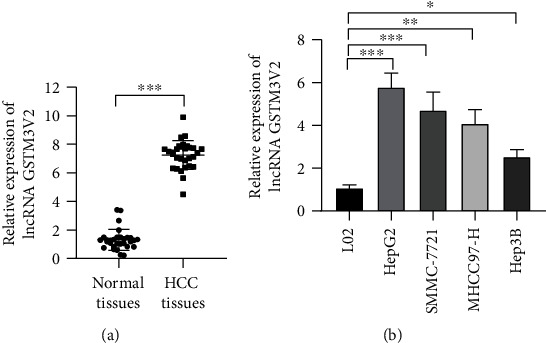
lncRNA GSTM3TV2 is increased in HCC tissues and HCC cell lines. (a) Real-time qPCR was performed to measure the level of lncRNA GSTM3TV2 between tumor tissues and adjacent normal tissues in HCC patients. (b) Real-time qPCR was used to identify lncRNA GSTM3TV2 level in LO2, HepG2, SMMC-7721, MHCC97-H, and HepG3B cells. Data are shown as mean ± SD. All experiments were repeated three times. ^∗^*P* < 0.5; ^∗∗^*P* < 0.01; ^∗∗∗^*P* < 0.001.

According to the study, it is the first time to measure the expression of lncRNA GSTM3TV2 by RT-qPCR method in 30 tumor tissues, compared with their adjacent normal tissues. Real time-PCR results indicated that lncRNA GSTM3TV2 was increased in tumor tissues significantly (Figure 1(a)). Furthermore, lncRNA GSTM3TV2 expression was detected in normal and HCC cell lines. We found that the lncRNA GSTM3TV2 expression level in HCC cell lines was about four times higher than that in normal cell lines (Figure 1(b)). The result pointed out that lncRNA GSTM3TV2 may function to be an oncogenic factor in HCC tissues and cell lines.

### 3.2. lncRNA GSTM3TV2 Functions as an Oncogenic Factor

**Figure 2:**
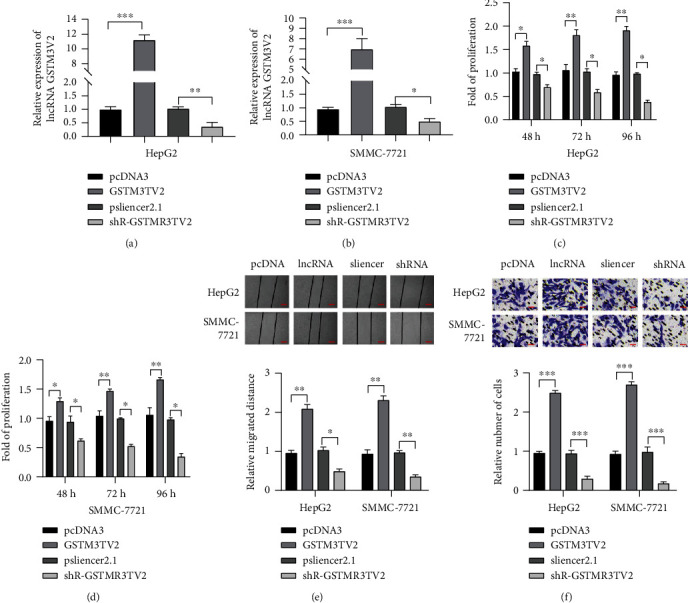
lncRNA GSTM3TV2 promotes proliferation, migration, and invasion in HCC cell lines. pcDNA3, pcDNA3/lncRNA GSTM3TV2, or pSilencer2.1/shR-lncRNA GSTM3TV2HCC were transfected into cell lines (HepG2 and SMMC-7721 cells). (a, b) Real-time qPCR was made to measure the level of lncRNA GSTM3TV2. (c, d) Cell viability was measured by MTT assay in HCC cell lines. (e, f) Wound healing assay and transwell assay were carried out to detect cell migration and invasion abilities. Data are shown as mean ± SD. All experiments were repeated three times. ^∗^*P* < 0.05; ^∗∗^*P* < 0.01; ^∗∗∗^*P* < 0.001. Scar bar = 100 *μ*m in wound healing assay. Scar bar = 20 *μ*m in a transwell assay.

Firstly, we constructed the overexpression plasmids of lncRNA GSTM3TV2 and its shRNAs to knock down its expression to investigate the expression of lncRNA GSTM3TV2 in HCC cell lines. Next, the gain-of-function and loss-of-function experiments were employed to overexpress or knock down the level of lncRNA GSTM3TV2 in HCC cell lines (HepG2 and SMMC-7721). RT-qPCR also demonstrated that the overexpression and knockdown of lncRNA GSTM3TV2 were workable (Figures 2(a) and 2(b)). Then, an MTT assay was performed to show that lncRNA GSTM3TV2 overexpression could promote the proliferation of HCC cells significantly, while decreased lncRNA GSTM3TV2 could reduce the cell viability markedly (Figures 2(c) and 2(d)). Furthermore, wound healing assay as well as transwell assay was employed to investigate whether lncRNA GSTM3TV2 could affect migration and invasion of HCC cell lines in the study. It showed that lncRNA GSTM3TV2 overexpression increased HCC cells migration and invasion significantly, while lncRNA GSTM3TV2 knockdown showed lower ability relatively (Figures 2(e) and 2(f)). Taken together, these results indicated that lncRNA GSTM3TV2 acted as an oncogenic factor, which could promote cell proliferation, migration, and invasion in HCC.

### 3.3. lncRNA GSTM3TV2 Sponges miR-597

**Figure 3:**
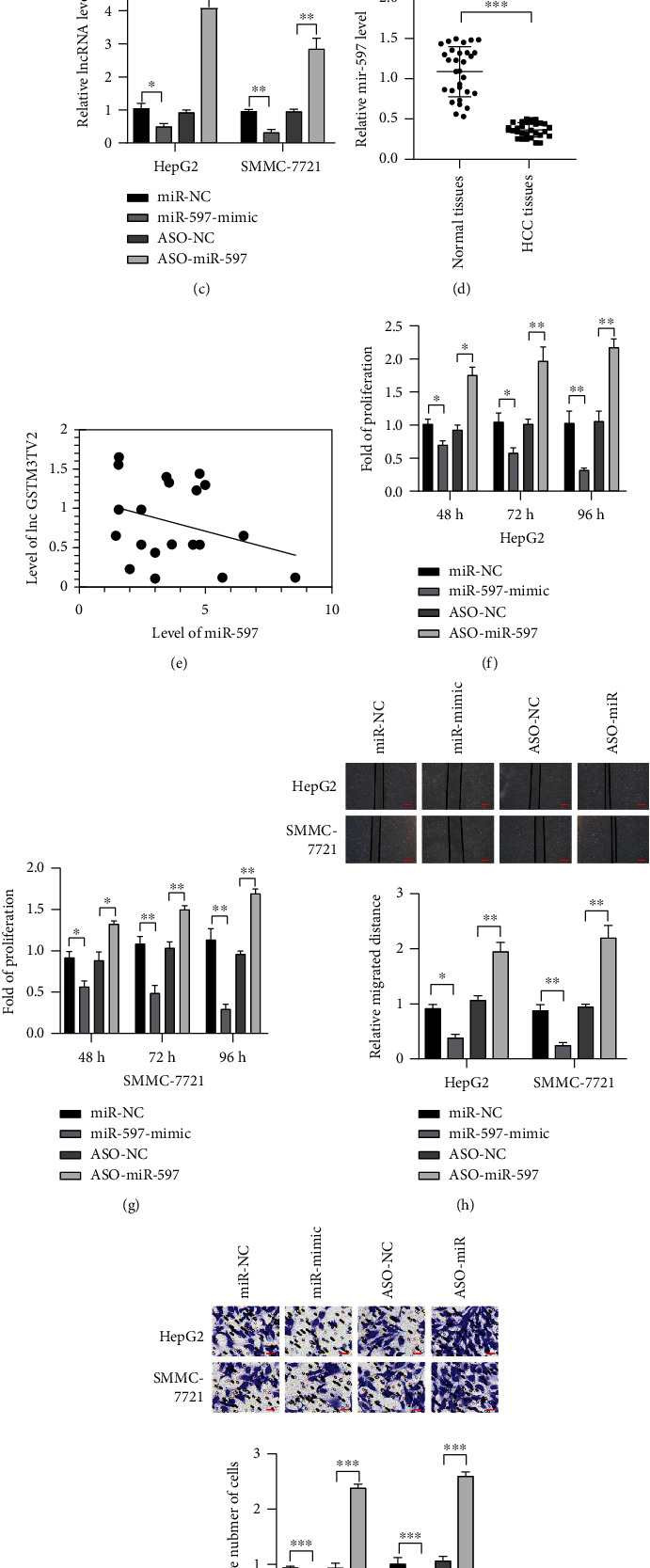
lncRNA GSTM3TV2 is targeted by miR-597. (a) The binding positions of miR-597 on lncRNA GSTM3TV2 were predicted by using miRDB, and the binding site of miR-597 on WT and the mutational lncRNA GSTM3TV2 were displayed. (b) Luciferase reporter system was constructed to measure the interaction between miR-597 and lncRNA GSTM3TV2. (c) The level of lncRNA GSTM3TV2 was measured by Real-time qPCR in HCC cells when transfecting miRNA mimics and ASO. (d) The level of miR-597 was measured by Real-time qPCR tumor and adjacent normal tissues in HCC patients. (e) The relationship between the expression of lncRNA GSTM3TV2 and miR-597 was analyzed by using Pearson's correlation analysis. (f, g) Cell viability was measured by MTT assay in HCC cell lines. (h, i) Wound healing assay and transwell assay were carried out to detect cell migration and invasion abilities. Data are shown as mean ± SD. All experiments were repeated three times. ^∗^*P* < 0.05; ^∗∗^*P* < 0.01; ^∗∗∗^*P* < 0.001. Scar bar = 100 *μ*m in wound healing assay. Scar bar = 20 *μ*m in a transwell assay.

More and more studies suggested that large numbers of lncRNAs can regulate the expression of the gene through acting as competing endogenous RNA (ceRNA). In order to convince whether lncRNA GSTM3TV2 could act as ceRNA to regulate gene expression, we used miRDB to predict the potential miRNA binding sites in lncRNA GSTM3TV2 (Figure 3(a)). miR-597 was chosen for further study because miR-597 is one of miRNA gaining high score. We also used luciferase assays to indicate that miR-597 could reduce luciferase activity when transfected with the wild-type lncRNA GSTM3TV2. However, miR-597 mimics did not affect the luciferase activity when cotransfected mutant lncRNA GSTM3TV2 into HCC cells (Figure 3(b)), which indicated that miR-597 could bind to lncRNA GSTM3TV2 directly. We also found that lncRNA GSTM3TV2 expression was downregulated transfecting with miR-597 mimics, while lncRNA GSTM3TV2 level was markedly upregulated after transfecting with miR-597 inhibitor (Figure 3(c)). At the same time, we detected the level of miR-597 in between HCC tissues and adjacent normal tissues, RT-PCR indicated that miR-597 was downregulated in HCC tissues (Figure 3(d)). Then, we used Pearson's correlation analysis to assess the relationship between the expression of miR-597 and lncRNA GSTM3TV2. It showed that the level of miR-597 was negative correlated with lncRNA GSTM3TV2 (Figure 3(e)). Next, we detected the role of miR-597 in HCC. We transfected the miR-597 mimics and ASO-miR-597 into HCC cell lines, and the results showed that overexpression of miR-597 could decrease the proliferation, migration, and invasion of HCC cell lines (Figures 3(f)–3(i)). Taken together, we demonstrated that lncRNA GSTM3TV2 could be bound to miR-597 directly.

### 3.4. MiR-597 Rescues the Tumor Phenotypes of lncRNA GSTM3TV2

**Figure 4:**
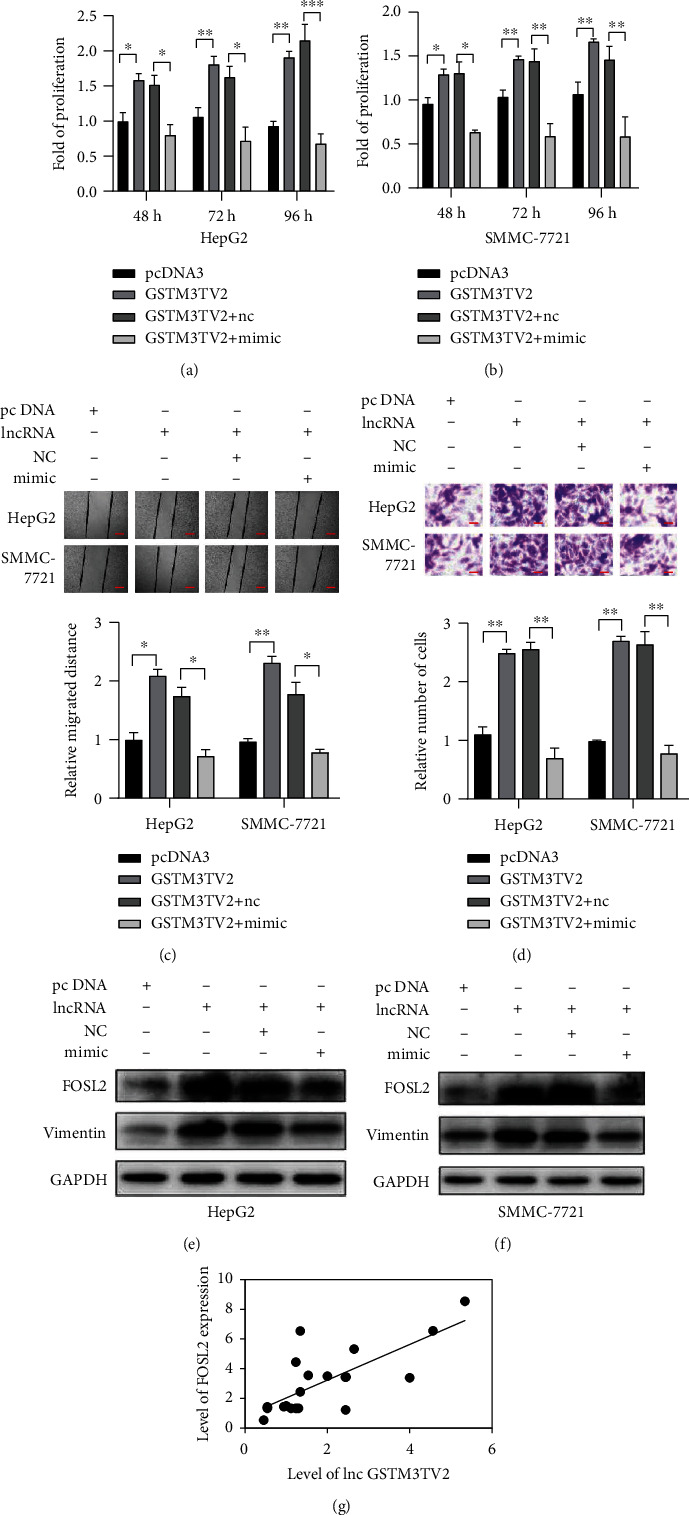
lncRNA GSTM3TV2 rescues the tumor phenotypes caused by miR-597. pcDNA3, lncRNA GSTM3TV2, lncRNA GSTM3TV2+miR-NC, and lncRNA GSTM3TV2+miR-597 mimics were cotransfected into HepG2 and SMMC-7721cells. (a–d) miR-597 mimics transfection could rescue viability, migration, and invasion in HCC cell lines. (e, f) FOSL2 and Vimentin protein levels were affected by lncRNA GSTM3TV2. (g) The relationship between the expression of lncRNA GSTM3TV2 and miR-597 was analyzed by using Pearson's correlation analysis. Data are shown as mean ± SD. All experiments were repeated three times. ^∗^*P* < 0.05; ^∗∗^*P* < 0.01; ^∗∗∗^*P* < 0.001. Scar bar = 100 *μ*m in wound healing assay. Scar bar = 20 *μ*m in a transwell assay.

Then, we conducted the rescue experiments to investigate that lncRNA GSTM3TV2 played its function through the level of miR-597 in HCC cells. The wound healing assays, as well as transwell assay, showed that cotransfection with miR-597 mimics could decrease the cell viability (Figures 4(a) and 4(b)), migration (Figure 4(c)), and invasion (Figure 4(d)), which were increased by lncRNA GSTM3TV2 by in HCC cells. Furthermore, we also investigated whether lncRNA GSTM3TV2 could have effects on the expression of FOSL2, one of the targets of miR-597. The results indicated that lncRNA GSTM3TV2 overexpression increased the level of FOSL2 and Vimentin. However, cotransfection with miR-597 mimics could rescue these effects in HCC cell lines (Figures 4(e) and 4(f)). Then, we used Pearson's correlation analysis to assess the relationship between lncRNA GSTM3TV2 and FOSL2. It showed that lncRNA GSTM3TV2 was positively correlated with the level of FOSL2 (Figure 4(g)). It indicated that lncRNA GSTM3TV2 acted as a sponge for miR-597 to increase the level of FOSL2, one of the miR-597 targets, to promote HCC cells carcinogenesis.

## 4. Discussion

Increasing studies have been shown that lncRNAs play a critical role in lots of cancers [9]. More and more evidence also suggested that lncRNA promoted carcinogenesis in cancers, such as pancreatic cancer [19], nonsmall cell lung cancer [20], and hepatocellular carcinoma [21]. A recent study showed that lncRNA GSTM3TV2 could promote pancreatic cancer gemcitabine resistance [18]. Meanwhile, we found that lncRNA GSTM3TV2 was also overexpressed in HCC tissues and HCC cell lines. Nevertheless, the function and mechanism of lncRNA GSTM3TV2 in HCC remains to uncover. We found that lncRNA GSTM3TV2 was upregulated in tumor tissues in HCC and HCC cell lines. In our study, it is the first time to show that lncRNA GSTM3TV2 can promote proliferation, migration, and invasion in HCC cell lines, further indicate the oncogenic role of lncRNA GSTM3TV2 in HCC. Furthermore, we used miRDB to predict the potential of miRNA binding sites to investigate the exact mechanisms of lncRNA GSTM3TV2 [22]. miR-597, one of the miRNA listed, was chosen for investigating further. It is also indicated that miR-597 could function as a tumor suppressor gene in lots of cancers. For instance, miR-597 promoted HCC progression by suppressing the expression of BRMS1 [23]. Another study showed that miR-597 promoted colorectal cancer cell proliferation in vitro and in vivo by targeting FBXL2 and activating the *β*-catenin signaling pathway [24]. However, it was also reported that miR-597 could suppress HCC cell proliferation dependent on SMYD3 in hepatocellular carcinoma [25]. So, the role of miR-597 in cancer is controversial now. Our study showed the downregulation of miR-597 in HCC could promote HCC cell proliferation, migration, and invasion.

We also performed luciferase reporter assays, showing that lncRNA GSTM3TV2 could be directly bound to miR-597, and decreased expression of miR-597. miR-597 was downregulated in HCC tissues and cell lines, presenting a negative correlation with lncRNA GSTM3TV2. Furthermore, we demonstrated that the relation between miR-597 and FOSL2 in HCC first time. We used the database to predict the target of miR-597, one of the highest scores. As we have known, miR-597 could act as a tumor suppressor in many cancer cells, and it could downregulate the FOSL2 to suppress breast cancer cell proliferation, migration, and invasion [26]. In our study, lncRNA GSTM3TV2 could downregulate the level of endogenous miR-597, thus, increasing the expression of FOSL2, demonstrating the ceRNA function of lncRNA GSTM3TV2 through sponging for miR-597. Thus, it is useful to detect the levels of lncRNAs, miRNAs, and other noncoding RNAs in the circulation at an early stage, and it may become a strategy to diagnose many cancers. In a word, we first demonstrate that lncRNA GSTM3TV2 is increased in tumor tissues cell lines in HCC. LncRNA GSTM3TV2 could function as an oncogene and promote HCC cell proliferation and migration by binding to miR-597, increasing the level of its target gene FOSL2. As we know, overexpression of lncRNA GSTM3TV2 increased HCC cell proliferation, migration, and invasion. Our findings indicate that lncRNA GSTM3TV2 plays an important role in HCC and can be used as a diagnostic biomarker and a target for HCC.

## Figures and Tables

**Table 1:** Clinicopathological features of HCC patients (*n* = 30).

Patients	*n* (%)
Age (years)	
<60	18 (60.0)
≥60	12 (40.0)
Gender	
Male	21 (70.0)
Female	9 (30.0)
T stage	
T1-T2	11 (36.7)
T3-T4	19 (63.3)
Regional lymph node metastasis	
Yes	22 (73.3)
No	8 (26.7)
Distance metastasis	
Yes	13 (43.3)
No	17 (56.7)
Tumor size	
<5 cm	17 (56.7)
≥5 cm	13 (43.3)

## Data Availability

The data used to support the findings of this study are included in the article.
